# A Challenging Cutaneous Lesion in a Patient With Chronic Idiopathic Neutropenia

**DOI:** 10.7759/cureus.21225

**Published:** 2022-01-14

**Authors:** Aikaterini Gkoufa, Pagona Sklapani, Nikolaos Trakas, Vasiliki E Georgakopoulou

**Affiliations:** 1 First Department of Internal Medicine, Laiko General Hospital, Athens, GRC; 2 Medical School, National and Kapodistrian University of Athens, Athens, GRC; 3 Department of Cytology, Mitera-Hygeia Hospital, Athens, GRC; 4 Biochemistry Department, Sismanogleio Hospital, Athens, GRC; 5 Department of Pulmonology, Laiko General Hospital, Athens, GRC; 6 First Pulmonology Department, Sismanogleio Hospital, Athens, GRC

**Keywords:** pseudomonas aeruginosa, cutaneous lesion, immunosuppression, neutropenia, ecthyma gangrenosum

## Abstract

Ecthyma gangrenosum (EG) is an uncommon necrotizing vasculitis that affects mainly immunocompromised and burn patients, and it is frequently associated with *Pseudomonas aeruginosa* bacteremia. However, cases of EG with other related pathogens and cases of EG affecting immunocompetent hosts have also been described in the literature. Besides, less common cases of EG without bacteremia have been reported. Herein, we describe a rare case of EG due to* Pseudomonas aeruginosa* without bacteremia in a patient with chronic idiopathic neutropenia (CIN). Considering the high mortality rate associated with EG, early diagnosis and appropriate effective treatment are crucial.

## Introduction

Ecthyma gangrenosum (EG) is a rare skin lesion first reported by Barker in 1897 [1]. It is often defined as a cutaneous manifestation of* Pseudomonas aeruginosa* septicemia; however, cases of EG that occurred without bacteremia have also been described [2]. Furthermore,* Pseudomonas aeruginosa* is not the only causative pathogen for these necrotic lesions, and various organisms may be identified [2].

The bacteremic type of EG is more common than the non-bacteremic one. In the bacteremic type, EG results from perivascular bacterial invasion of the media and adventitia of arteries and veins, with secondary ischemic necrosis of the surrounding skin. In the non-bacteremic type, the lesions are formed at the location of direct inoculation into the epidermis [2]. EG commonly begins as painless red macules that rapidly evolve into pustules and/or bullae. Ultimately, these become gangrenous ulcers [3].

The common risk factors for EG development include neutropenia, leukemia, diabetes mellitus, malnutrition, infancy, and old age, because of their underdeveloped or compromised immune system, recent viral illness, hypogammaglobulinemia, malignancies, and extensive burn wounds [4]. Ecthyma gangrenosum can occur in all age groups and genders, and although the majority of the cases are described in immunocompromised hosts, EG has also been described in immunocompetent individuals [5].

Chronic idiopathic neutropenia (CIN) in adults is an uncommon neutrophil disorder characterized by the continuous and unexplained decreased peripheral blood neutrophil count for a prolonged time period in the absence of evidence of any existing condition that might be associated with neutropenia and in the context of negative antineutrophil antibody testing and normal bone marrow (BM) morphology and karyotype, which exclude cases of myelodysplastic syndrome presenting as isolated neutropenia [6]. The pathophysiologic mechanism of CIN is related to an inflammatory BM microenvironment. This microenvironment consists of pro-inflammatory cytokines that induce myelosuppression, leading to the accelerated apoptosis of the granulocytic progenitor cells [7].

Herein, we report a rare case of EG without bacteremia in a patient with CIN.

## Case presentation

**Figure 1:**
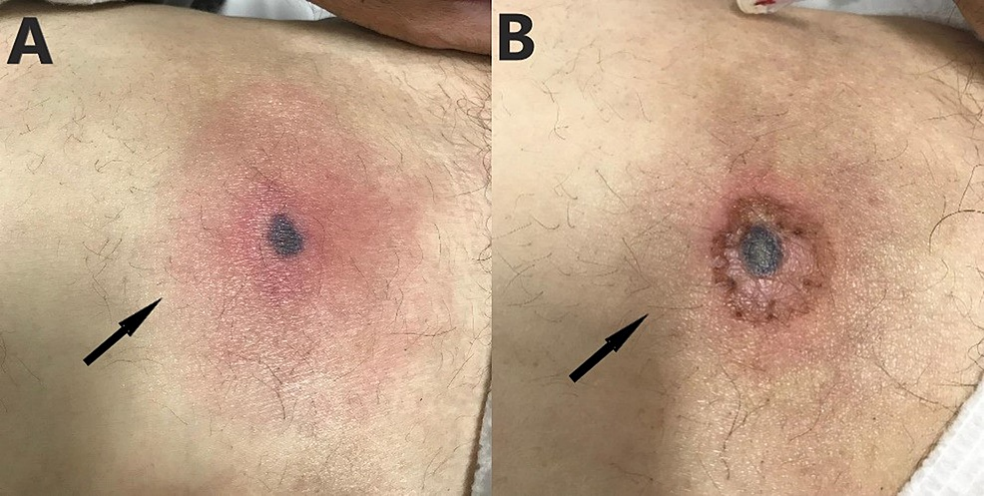
Ecthyma gangrenosum in a patient with chronic idiopathic neutropenia. A: Ecthyma gangrenosum at initial presentation; skin lesion of the abdominal wall with an erythematous halo surrounding a dark gray necrotic nodule (arrow). B: Ecthyma gangrenosum after successful treatment (arrow).

A 67-year-old male with a medical history of CIN and colonization of a multidrug-resistant *Pseudomonas *spp. was admitted to the emergency department because of a fever of up to 38.5°C of two days duration. On admission, the patient complained of a new-onset painful skin lesion of the abdominal wall with an erythematous halo surrounding a dark gray necrotic nodule (Figure 1A). He was hemodynamically stable and febrile.

The clinical examination revealed the already known splenomegaly. Laboratory analysis showed low white blood cell count, with low neutrophil count, and elevated levels of C-reactive protein (CRP). In addition, blood cultures and cultures of the skin lesion were obtained.

An antimicrobial treatment with intravenous colistin at a loading dose of 9.000.000 IU followed 12 hours later by a dose of 4.500.000 IU every 12 hours combined with intravenous gentamycin 240 mg once daily and intravenous vancomycin 1 gr twice daily was administrated.

*Pseudomonas aeruginosa* was isolated from the ulcer’s culture; however, numerous blood cultures were sterile. Three days after the patient’s presentation, the susceptibility testing of *Pseudomonas aeruginosa* was available, so antimicrobial treatment was deescalated to intravenous piperacillin/tazobactam 4/0.5 g four times daily, and on the fourth day of hospitalization, the patient remained afebrile. After eight days of intravenous antimicrobial treatment with piperacillin/tazobactam and clinical improvement, the patient was discharged and was advised to continue his treatment with oral ciprofloxacin for seven days until reevaluation. Figure 1B shows the improvement of the skin lesion after successful treatment.

## Discussion

Our case is rare for two reasons. Firstly, it describes a case of EG without bacteremia, which is the less common type of EG. Secondly, to the best of our knowledge, our case is the first to report the presence of EG due to *Pseudomonas aeruginosa* in a patient with CIN.

This skin lesion without bacteremia has been described in heart, kidney, and bone marrow transplant recipients [8-10]. It has also been described in neutropenic patients [11]. Furthermore, it has been reported in patients with various types of immunosuppression, such as human immune virus (HIV) infection and Good syndrome, a rare association of thymoma and immunodeficiency [12,13]. EG without bacteremia has been also mentioned in leukemic patients [14]. Besides, EG without bacteremia due to *Pseudomonas aeruginosa* has been described in previously healthy individuals [15,16]. Of interest, according to a case series by Huminer et al., the most important difference between patients with EG with bacteremia and patients with EG without bacteremia was a significantly lower mortality rate in non-bacteremic patients [11].

CIN is a relatively rare disorder. In a study by Andersen et al., among a Danish health service database of 370,000 individuals, the prevalence of CIN was 0.12% [17]. In another study from the island of Crete, in our country, the prevalence of CIN in adults was 1.4%, with no cases of severe neutropenia among 778 adults [18]. In Washington, USA, the estimated prevalence of severe CIN is approximately five cases per million [6]. For several years, there was no effective treatment for CIN. Numerous studies have now confirmed the effectiveness of granulocyte colony-stimulating factor (G-CSF) for the treatment of severe CIN and for the reduction of infection-related events and antibiotic use [19].

However, it has been reported that these patients are mainly vulnerable to respiratory, periodontal, and skin infections and oral ulcers, despite receiving G-CSF [6]. Thus, the role of early clinical suspicion of the diagnosis of EG may be crucial due to its rarity and taking into account that the mortality rate of EG ranges from 38% to 96% in cases with bacteremia and from 7% to 15% in non-bacteremic patients [20].

## Conclusions

This is a rare case of EG without bacteremia in a patient with CIN. The case indicates that EG may develop even in the absence of bacteremia in this patients’ group, and it should be suspected as a possible diagnosis even in the absence of positive blood cultures. Considering the high mortality rate, early recognition and appropriate effective treatment are mandatory.
